# Bacteriological profile and antibiotic susceptibility of neonatal sepsis in neonatal intensive care unit of a tertiary hospital in Nepal

**DOI:** 10.1186/s12887-018-1176-x

**Published:** 2018-06-27

**Authors:** Bhishma Pokhrel, Tapendra Koirala, Ganesh Shah, Suchita Joshi, Pinky Baral

**Affiliations:** 1 Department of Pediatrics, Patan Academy of Health Sciences, Lagankhel, PO Box 26500, Lalitpur, Nepal; 2 School of Medicine, Patan Academy of Health Sciences, Lagankhel, Lalitpur, Nepal; 3 School of Health and Allied Sciences, Pokhara University, Lekhnath-12, Kaski, Nepal

**Keywords:** Antibiotic susceptibility, Klebsiella, Multi-drug resistance, Neonatal sepsis, NICU

## Abstract

**Background:**

Neonatal sepsis, one of the leading causes of mortality in neonatal intensive care units (NICU) of developing countries like Nepal, is often not extensively studied. In order to decrease the morbidity and mortality associated with neonatal sepsis, neonatologists should have a keen knowledge of the existing bacteriological flora and their antibiotic susceptibility pattern. In this study, we aim to determine the bacteriological profile and antibiotic susceptibility pattern of culture positive neonatal sepsis in the NICU of a tertiary teaching hospital in Nepal.

**Methods:**

This was a retrospective cross-sectional study of all blood culture positive sepsis cases among neonates admitted to the neonatal intensive care unit of Patan Hospital, Nepal between April 15, 2014 and April 15, 2017. All neonates with a clinical suspicion of sepsis with a positive blood culture were identified. Patient demographics, clinical details, maternal risk factors, and laboratory data including bacteriological profiles and antimicrobial susceptibilities were recorded and analyzed.

**Results:**

Of the 336 neonates admitted in the NICU, 69 (20.5%) had culture-positive sepsis. The majority were early-onset sepsis (*n* = 54, 78.3%) and were among the preterm babies (*n* = 47, 68.1%). Most bacterial isolates were gram-negative, predominantly the Klebsiella species (*n* = 23, 33.3%). Klebsiella showed high resistance to commonly used antibiotics such as; Cefotaxime (90.5%), Gentamicin (75%), Ciprofloxacin (76.2%), Ofloxacin (72.2%) and Chloramphenicol (65%). However, they showed good susceptibility to Carbapenems (100%), Colistin (88.8%) and Tigecycline (81.8%). Among cultures with gram-positive species, Coagulase-negative Staphylococci (CONS) (*n* = 14, 20.3%) predominated. CONS showed high resistance to Oxacillin (80%), Cefotaxime (66.7%) and Meropenem (80%) but good susceptibility (100%) to Vancomycin and Linezolid. Prevalence of multidrug-resistant strain was 73.9%.

**Conclusions:**

Klebsiella species and CONS were the most common causes of neonatal sepsis in our study. A significant proportion of the isolates were multidrug resistant strains, which pose a great threat to neonatal survival, and thereby, warrant modification of existing empirical therapy. Implementation of effective preventive strategies to combat the emergence of antibiotic resistance is urgently needed. We recommend a combination of Piperacillin-Tazobactam and Ofloxacin as the first line therapy and combination of Vancomycin and Meropenem as the second line empirical therapy in our NICU.

## Background

Sepsis is considered one of the leading causes of neonatal mortality globally, more so in developing countries like Nepal [1]. According to Nepal Demographic and Health Survey 2016, national neonatal mortality rate was 21 per thousand live births. Infections including sepsis contributed to 16% of the neonatal mortality [2]. Emergence of antimicrobial resistance has become a global concern. With a limited reserve of antibiotics, increasing antimicrobial resistance has become a great challenge in the management of neonatal sepsis. Knowledge of prevalent bacterial isolates and their antibiotic susceptibility pattern is crucial when choosing the appropriate empirical therapy in order to decrease morbidity and mortality. There is, however, a paucity of such data in Neonatal Intensive Care Units (NICU) of Nepal. We aim to determine the prevalence of culture-positive neonatal sepsis, its clinico-bacteriological profile and antibiotic susceptibility pattern in the NICU of Patan Hospital, Lalitpur, Nepal.

## Methods

This was a retrospective cross-sectional study conducted in the NICU of Patan Hospital. Patan Hospital is the tertiary level teaching hospital of Patan Academy of Health Sciences (PAHS) located in Lalitpur, Nepal. It has a six-bed NICU, caring on average for 120 critically ill neonates annually. Neonates admitted to the NICU between April 15, 2014 and April 15, 2017 with clinical features of sepsis and who had a positive blood culture were included in the study. Blood cultures were sent in neonates with either a clinical suspicion of sepsis or risk factors for it. Sepsis was suspected in the presence of temperature instability, lethargy, feeding intolerance, respiratory distress, hemodynamic instability, convulsion, hypotonia, irritability or bleeding diathesis. Prematurity (< 37 weeks of gestation), low birth weight (< 2500 g), history of resuscitation at birth, rupture of membrane for more than 18 h (PROM), antepartum fever, foul-smelling liquor and repeated (≥3) unclean per vaginal examinations were considered as risk factors for neonatal sepsis.

Patan Hospital follows standard microbiological techniques. Before drawing blood, the skin is disinfected with 10% Povidone-iodine solution for 2 min, followed by 0.5% Chlorhexidine solution for 1 minute. One to three milliliters of blood is taken aseptically from a peripheral vein and injected into the BACTEC PedsPlus™(Becton Dickinson, Ireland) culture vials. It is then incubated in an automated BACTEC system at 35 ± 2 °C for 5 days as per manufacturer’s instructions. Subculture and organism identification is performed as described by Koneman et al. [3]. Antibiotic susceptibility test is done using the Kirby-Bauer disc diffusion method, as per the Clinical and Laboratory Standards Institute (CLSI) guidelines (2014) [4]. After collection of blood for culture, neonates are started on empiric intravenous Ampicillin and Amikacin (first line therapy). If there is no clinical response after 48–72 h, antibiotics are upgraded to intravenous Chloramphenicol and Ofloxacin (second line) or Meropenem and Colistin (third line). These are later modified, based on culture and antibiotic susceptibility results. Coagulase-negative Staphylococcus (CONS) isolated from non-septic neonates, in whom the repeat culture showed no growth, was considered as a contaminant and hence excluded from the study. Early-onset sepsis (EOS) was defined as sepsis occurring within first 72 h of life, that occurring after 72 h of life was defined as late-onset sepsis (LOS) [5]. Multidrug-resistant (MDR) strains were defined as per international standard definitions for acquired resistance and relative to the panel of antibiotics tested for each isolate, as in vitro non-susceptibility to ≥1 agent in ≥3 antimicrobial categories: Penicillins, Cephalosporins, Beta-lactamase inhibitor combinations, Fluoroquinolones, Aminoglycosides, Chloramphenicol, Folate pathway inhibitors, Tetracyclines, Macrolides and Glycopeptides [6].

For data collection, microbiology laboratory blood culture registers were reviewed and all blood culture positive neonates were identified. Their records were subsequently evaluated for clinical evidence of sepsis and enrolled in the study. Data on age at admission, gestational age at birth, birth weight, maternal risk factors, laboratory parameters, blood culture isolates and their susceptibility pattern and clinical outcome were collected. EpiInfo™ for Mobile was used for data entry and Statistical Package for Social Sciences (SPSS) version 21 was used for data analysis. Summary of measures were reported as percentage for categorical variables and as mean with standard deviation for quantitative variables. Fisher’s exact test was used to infer any differences between the categorical variables and *p*-value of less than 0.05 was considered statistically significant. Ethical approval to conduct the study was obtained from the Institutional Review Committee (IRC) of PAHS.

## Results

### General characteristics and clinical profile

During the study period, 24,516 live births occurred, and 336 neonates were admitted in our NICU of whom 332 had their blood sent for culture and susceptibility test. Out of 336 neonates, 69 (20.5%) had culture-positive sepsis. EOS was found in 78.3%. Among neonates with positive cultures, 63.8% had a birth weight less than 2500 g, 68.1% were preterm and 27.5% were delivered by emergency cesarean section (Table 1). Forty-five percent had a maternal history of PROM, which was more common among the EOS group (54%). Maternal Group B Streptococcus (GBS) colonization status was unknown in 69.6%.

**Table 1:** General characteristics of the enrolled neonates

Variables	EOS group	LOS group	Total	Percent	Fisher’s exact test *p*-value
Neonatal variables					
Gender					
Male	31	6	37	53.6	0.2571
Female	23	9	32	46.4	
Gestational age at birth					
Preterm (< 37 weeks)	42	5	47	68.1	0.0033
Term (> 37 weeks)	12	10	22	31.9	
Birthweight					
< 2500 g	34	10	44	63.8	1.0000
≥ 2500 g	20	5	25	36.2	
Mode of delivery					
Vaginal	35	8	43	62.3	0.5484
Caesarean section	19	7	26	37.7	
APGAR score < 6 at 5 min	5	0	5	7.3	0.6250
Maternal variables					
Maternal fever (within 7 days before delivery)	9	0	9	13.0	0.0039
PROM of > 18 h	29	2	31	44.9	< 0.0001
Foul smelling liquor	2	0	2	2.9	0.5000
Maternal antibiotics (within 7 days before delivery)	19	2	21	30.4	0.0002
Maternal GBS colonization	12	0	12	17.4	0.0004
Neonatal care related variables					
Need for inotropes	39	2	41	59.4	< 0.0001
Need for positive pressure ventilation	54	10	64	92.8	< 0.0001
Central line	47	3	50	72.5	< 0.0001
Mortality	11	0	11	15.9	0.0009

*EOS* Early onset sepsis, *GBS* Group B Streptococcus, *LOS* Late onset sepsis, *PROM* Prolonged rupture of membrane

The common clinical findings observed at admission were respiratory distress (79.7%), tachycardia (60.9%), cyanosis (59.4%) and hypothermia (53.6%). Similarly, low absolute neutrophil count (ANC) (< 1800/mm^3^), thrombocytopenia (< 150,000/mm^3^) and raised C-reactive protein (CRP) (> 10 mg/dl) were seen in 20, 75 and 84% respectively. During the course of treatment, feeding intolerance, seizure, and dysglycemia (blood sugar level < 40 mg/dl requiring dextrose bolus or > 250 mg/dl requiring insulin infusion) was observed in 46.4, 31.9 and 27.5% respectively. The mean duration of NICU stay was 16.0 ± 10.7 days and the mortality rate was 15.9%.

### Bacteriological profile

The majority of bacterial isolates were gram-negative (77%). Among the total isolates, Klebsiella species, CONS and Enterobacter were the most common (Table 2). Five cases (7.24%) had polymicrobial sepsis of which two had yeast cells along with bacterial growth.

**Table 2:** Distribution of bacterial isolates with their relative frequency

Bacterial isolate	Number	Percent
Gram-negatives		
Klebsiella species	23	33.3
Enterobacter species	13	18.8
Acinetobacter species	8	11.6
*Escherichia coli*	3	4.3
*Serratia rubidaea*	3	4.3
Pseudomonas species	2	2.9
Bacillus species	1	1.4
Gram-positives		
CONS	14	20.3
*Staphylococcus aureus*	1	1.4
Non-hemolytic streptococcus	1	1.4
Total	69	100.0

CONS Coagulase negative staphylococci

Klebsiella, CONS and Enterobacter species were the most common organisms found in all groups; in both EOS and LOS, term and preterm babies. There was preponderance among EOS and preterm infants (Table 3); however, this observed difference was not statistically significant (*p*-value> 0.05) except for Klebsiella in EOS group (*p*-value 0.0025.

**Table 3:** Distribution of isolates based on age at admission and gestational age at birth

Bacterial isolate	Age at admission			Gestational age at birth		
	< 72 h (EOS)	> 72 h (LOS)	Fisher’s exact test (*p*-value)	Pre-term	Term	Fisher’s exact test (*p*-value)
Klebsiella	19	4	0.0025	15	8	0.2100
CONS	11	3	0.0573	8	6	0.7905
Enterobacter	9	4	0.2668	10	3	0.0922
Acinetobacter	6	2	0.2890	5	3	0.7265
*Serratia rubidaea*	3	0	0.2500	2	1	1.0000
*Escherichia coli*	2	1	1.0000	3	0	0.2500
Pseudomonas	2	0	0.5000	2	0	0.5000
Bacillus	1	0	1.0000	1	0	1.0000
*Staphylococcus aureus*	1	0	1.0000	1	0	1.0000
Non-hemolytic streptococcus	0	1	1.0000	0	1	1.0000
Total	54	15		47	22	

*CONS* Coagulase negative staphylococcus, *EOS* Early onset sepsis, *LOS* Late onset sepsis

### Antibiotic susceptibility pattern

#### Among gram-negative organisms

Within the beta-lactam antibiotics, Klebsiella demonstrated maximum susceptibility to Meropenem (100%), Imipenem (100%) and Piperacillin-Tazobactam (Pip-Taz) (60%) while showing high resistance to Ampicillin-Sulbactam (66.7%) and Cefotaxime (90.5%). Among non-beta-lactam antibiotics, Klebsiella showed maximum susceptibility to Colistin (88.8%) and Tigecycline (81.8%) while showing high resistance to Aminoglycosides and Quinolones.

Enterobacter species demonstrated high susceptibility to Meropenem (80%), Tigecycline (85.7%) and Colistin (87.5%) while demonstrating high resistance to Cefotaxime (83.4%).

Acinetobacter demonstrated good susceptibility to Ciprofloxacin (81.2%), Colistin (80%) and Tigecycline (66.7%) while it was highly resistant to Amikacin (100%), Chloramphenicol (100%) and Cefotaxime (85.7%).

*Escherichia coli* demonstrated marked resistance to commonly used antibiotics, showing susceptibility only to reserved antibiotics like Tigecycline and Colistin (Table 4).

**Table 4:** Antibiotics resistance among the major isolates

Antibiotic	Klebsiella (N = 23)		CONS (14)		Enterobacter (*N* = 13)		Acinetobacter (*N* = 8)		*Escherichia coli* (*N* = 3)		*Serratia rubidaea* (N = 3)	
	R/(R + S)	R %	R/(R + S)	R %	R/(R + S)	R %	R/(R + S)	R %	R/(R + S)	R %	R/(R + S)	R %
Beta-lactam Antibiotics												
Oxacillin	6/6	100	8/10	80	3/3	100	2/2	100	1/1	100	1/2	50
Cefotaxime	19/21	90.5	4/6	66.7	10/12	83.4	6/7	85.7	3/3	100	1/3	33.3
Meropenem	0/18	0	4/5	80	2/10	20	4/7	57.1	2/3	66.7	0/3	0
Pip-Taz	4/10	40	1/2	50	2/4	50	3/6	50	2/2	100	1/3	33.3
Non-beta-lactam Antibiotics												
Amikacin	12/21	57	5/10	50	0/1	0	7/7	100	3/3	100	1/3	33.3
Gentamicin	15/20	75	7/12	58.3	5/13	38.5	5/7	71.4	2/3	66.7	1/3	33.3
Chloramphenicol	13/20	65	5/11	45.5	8/13	61.5	7/7	100	3/3	100	3/3	100
Ciprofloxacin	16/21	76.2	8/10	80	5/13	38.5	3/16	18.8	3/3	100	1/3	33.3
Ofloxacin	13/18	72.2	8/12	66.7	1/12	8.3	4/6	66.7	3/3	100	1/3	33.3
Linezolid	–	–	0/3	0	–	–	1/1	100	–	–	0/1	0
Vancomycin	1/1	100	0/4	0	1/1	100	–	–	–	–	0/1	0
Tigecycline	2/11	18.2	–	–	1/7	14.3	2/6	33.3	0/2	0	0/1	0
Colistin	2/18	11.2	–	–	1/8	12.5	1/5	20	0/3	0	1/3	33.3

*CONS* Coagulase negative staphylococi, *Pip-Taz* Piperacillin-Tazobactam, *R* Number of resistant isolates, *R%* Percentage of resistant isolates, *S* Number of susceptible isolates, [−] Not tested

#### Among gram-positive organisms

CONS, Methicillin-resistant *Staphylococcus aureus* (MRSA) and Non-hemolytic Streptococcus were the most common gram-positive organisms associated with neonatal sepsis in our study. The majority of CONS were resistant to commonly used antibiotics (Table 4). A single case of MRSA isolated in our study showed susceptibility to Amikacin, Gentamicin, Ofloxacin, Pip-Taz, and Linezolid. One case of Non-hemolytic Streptococcus isolate showed susceptibility to Amoxicillin, Gentamicin and Chloramphenicol, but surprisingly resistance to Cefotaxime and Ofloxacin.

#### Status of global antibiotic resistance

Overall resistance to individual antibiotics among gram-positive and gram-negative isolates is summarized in Table 5. It shows alarming rates of resistance to commonly used antibiotics. The resistance to the current first and second line empirical therapy was 72 and 65% respectively.

**Table 5:** Overall status of antibiotic resistance among the gram-positive and gram-negative isolates

Antibiotics tested	Gram-negative		Gram-positive	
	R	R %	R	R %
Beta-lactam antibiotics				
Amoxicillin	38	100.0	7	87.5
Oxacillin	11	91.7	8	100.0
Cefotaxime	33	80.5	5	62.5
Meropenem	4	11.8	2	40.0
Piperacillin-Tazobactam	9	47.4	1	33.3
Ampicillin-Sulbactam	2	66.7	1	100.0
Imipenem	1	8.3	–	–
Aztreonam	1	50.0	–	–
Non-beta-lactam antibiotics				
Amikacin	21	50.0	5	45.5
Gentamicin	24	60.0	7	50.0
Tobramycin	1	50.0	1	100.0
Chloramphenicol	29	70.7	6	46.2
Ciprofloxacin	25	62.5	8	61.5
Ofloxacin	18	47.4	9	64.3
Linezolid	–	–	0	0.0
Vancomycin	2	66.7	0	0.0
Tigecycline	3	14.3	–	–
Cotrimoxazole	1	33.3	–	–
Colistin	6	17.6	3	100.0
Teicoplanin	1	50.0	–	87.5

*R* Number of resistant isolates, *R%* Percentage of resistant isolates

## Discussion

Neonatal sepsis is considered the leading cause of infant mortality and morbidity in the NICU. Two previous studies conducted in neonatal nurseries from Patan Hospital during the period of 2000–2005 and 2006–2007 showed culture positivity of 13.7 and 19.56% respectively [7, 8]. However, our study, which is first of its kind to be conducted in NICU of the same institute, showed culture positivity of neonatal sepsis to be 20.7%. In contrast, studies conducted at KIST Medical College and Manipal College of Medical Sciences, Nepal showed culture positivity to be 48 and 44.9% respectively [9, 10]. Variations in culture positivity rate of neonatal sepsis in different studies seem to arise from differences in culture-techniques and study designs.

The majority of culture positive sepsis was EOS and among preterm and low birth weight neonates, similar to the study findings of Kathmandu University Hospital (KUH), Dhulikhel, Nepal [11].

The most common clinical manifestation of neonatal sepsis in our study was respiratory distress (79%). Similar findings were noted in studies from KIST Medical College, Nepal (54%) and Beni Suef University Hospital, Egypt (36%) [9, 12]. At our center, we take CRP as a biomarker of sepsis and its serial decline is taken as laboratory evidence of improvement. In the initial screening test, the majority had raised CRP (75%) and low platelet count (84%) whereas low ANC was seen only in 20% of the cases.

The majority of the isolates were gram-negative, similar to the findings of Shrestha S et al. and that of investigators of the Delhi Neonatal Infection Study (DeNIS) Collaboration [11, 13]. In contrast, Peterside O et al. in Nigeria and Sharma P et al. in India showed a preponderance of gram-positive organisms of which *Staphylococcus aureus* was the most prevalent [14]. One reason for this variation could be due to the difference in adherence to infection prevention and control measures.

Klebsiella species were the most frequent causative organisms of neonatal sepsis in our study, a similar finding to that of Shrestha S et al. [11]. In contrast, previous studies conducted at the same institute in the neonatal nurseries showed CONS as a major isolate [7, 8]. The variation in the major isolate could be due to differences in study setting, study population and adherence to hand hygiene practices. Similar CONS predominance was reported by Mohamadi P et al. [15]. The same bacterial isolates were attributed to neonatal sepsis among the EOS and LOS groups, in agreement with Shrestha S et al’s and Singh HK et al’s [11, 16] findings. In contrast, studies by Mahmood A et al. and Ingale HD et al. demonstrated Klebsiella in EOS and Staphylococcus in LOS as common causative organisms [17, 18]. Wu JH et al. in Taiwan, found GBS and Methicillin resistant-CONS to be the most frequent cause among EOS and LOS respectively [19].

Our study shows the majority of causative organisms have developed resistance to these frequently used antibiotics; Amoxicillin, Cefotaxime and Oxacillin from the beta-lactam group. This finding is consistent with studies done in neonatal nurseries of the same institute and NICUs in other parts of Nepal and Pakistan [7, 8, 11, 12, 17]. Both gram-positive and gram-negative organisms showed high susceptibility to Carbapenems, a similar finding to other studies conducted both inside and outside Nepal [11, 12, 17]. Similarly, gram-negative organisms showed high susceptibility to Colistin, which is consistent with the findings of Jessan Bonny et al. [20].

Vancomycin and Linezolid showed high (100%) susceptibility towards gram-positive isolates, similar to the finding’s of Mullah SA et al. and Singh HK [16, 21]. Amikacin showed moderate susceptibility against both gram-positive and negatives. Among second-line antibiotics, Chloramphenicol had low susceptibility (29.3%) against gram-negatives compared to gram-positives (53.8%). Whereas Ofloxacin had moderate susceptibility (52.6%) to gram-negatives.

Klebsiella and Enterobacter, the main gram-negative isolates showed maximum susceptibility to Carbapenems, followed by Colistin and Tigecycline respectively. Such high susceptibility toward Carbapenem was also documented by Sheth KV et and Yusuf D et al. [22, 23].

Acinetobacter demonstrated good susceptibility to Ciprofloxacin, Colistin, and Tigecycline. Although our study showed high susceptibility towards Ciprofloxacin various other studies reported low susceptibility [11, 24].

*Escherichia coli* showed high resistance to the first and second line empirical antibiotics used commonly in our institution, only demonstrating susceptibility towards Colistin and Tigecycline. In contrast to this, Singh HK et al. and Sheth KV et al. showed good susceptibility towards commonly used antibiotics [16, 24]. This indicates the emergence of highly resistant strains of *Escherichia coli* in our setting.

CONS has been reported in various studies as the most common cause of neonatal sepsis in NICUs [19, 22]. The second commonest cause of neonatal sepsis in our study, CONS showed low susceptibility to Penicillin, third generation Cephalosporin and intermediate to Aminoglycosides and high susceptibility to Linezolid and Vancomycin. Sarangi KK et al. and Dalal P et al. also demonstrated high Vancomycin susceptibility in their studies [25, 26].

GBS, the most common cause of EOS in high-income countries, has a low reported incidence in low and middle-income countries [27]. Such low incidence of GBS sepsis in EONS is consistent with our findings. Possible reasons for this could include overuse of antibiotics during the antenatal period or substandard culture techniques and microbiological methods [28, 29]. At our institution, intravenous Crystalline Penicillin is given for mothers with PROM and intravenous Metronidazole and Gentamicin along with Crystalline Penicillin for mothers with chorioamnionitis as intrapartum antibiotic prophylaxis. Over diagnosis of PROM and chorioamnionitis and subsequent antibiotic treatment could be the reason for low yield of GBS at our institution.

In our study, the overall mortality rate in culture positive sepsis was 15.94%, which is consistent with the studies from Egypt and India [12, 30, 18]. The highest mortality was seen in the Enterobacter and Klebsiella sepsis group. Though the highest case fatality rate was observed with Pseudomonas sepsis, its limited yield hinders the generalization of this result. A combination of Pip-Taz and Ofloxacin as first line empirical therapy, or Vancomycin and Meropenem as second line would reduce the overall resistance by 22 and 46% respectively. The current first line therapy covers only 28% of the isolates whereas the proposed first line therapy with Pip-taz and Ofloxacin would successfully cover 50% of the isolates.

The emergence of MDR bacteria presents a great challenge to the management of neonatal sepsis, causing significant morbidity and mortality. The prevalence of neonatal sepsis due to MDR strains in our study was 73.91%. MDR among gram-negatives and gram-positives was 80.76 and 52.94% respectively in our study, which is in agreement with the findings of DeNIS Collaboration from India and Labi AK et al. from Ghana [13, 6].

The retrospective design of our study, together with its single centered, small study population and limited yield of some pathogens were all limitations in our study. Hence, large-scale, multi-center prospective studies are needed to validate our findings.

## Conclusions

Our study revealed gram-negative isolates as the predominant pathogens in both EOS and LOS groups. Both gram-positive and gram-negative isolates showed high resistance to commonly used antibiotics. Significant proportions of them were MDR strains. Such high antibiotic resistance is associated with significant neonatal morbidity and mortality. Based on our findings, a combination of Pip-Taz and Ofloxacin as first line therapy, or a combination of Vancomycin and Meropenem as second line would be the appropriate empirical therapy. However, the use of the broad-spectrum antibiotics as empirical therapy could be detrimental in the long run and hence they should be used judiciously and modified to narrow spectrum antibiotics, as guided by the culture and susceptibility report at the earliest opportunity. The best prevention of neonatal sepsis comprises of early recognition of high-risk infants and strict infection control practices, such as safe delivery, hand hygiene, avoidance of unnecessary invasive procedures and restricted entry to the NICU. To prevent the emergence of drug resistance, comprehensive approach consisting of evaluation of antibiotic consumption, improvement in laboratory techniques, rational use of empirical therapy and de-escalation/discontinuation of therapy when suitable along with continuous surveillance and monitoring of local epidemiology is needed. Use of synbiotics in a recent trial in India has shown promising results in prevention of neonatal sepsis in developing countries [31]. Use of Matrix assisted laser deserption ionization-time of flight mass spectrometry (MALDI-TOF MS), a nobel technique for the rapid identification of isolates and their antimicrobial susceptibility is yet to be explored in low-income countries like Nepal.

## Abbreviations

ANC: Absolute neutrophil count
CONS: Coagulase-negative Staphylococci
CRP: C-reactive protein
EOS: Early-onset sepsis
GBS: Group B Streptococcus
LOS: Late-onset Sepsis
MDR: Multidrug-resistant
MRSA: Methicillin-resistant *Staphylococcus aureus*
NICU: Neonatal intensive care unit
PIP-TAZ: Piperacillin-Tazobactam
PROM: Prolonged rupture of membrane
